# Spinal Cord Stimulation for Functional Restoration in Spinal Cord Injury: A Narrative Review

**DOI:** 10.7759/cureus.78610

**Published:** 2025-02-06

**Authors:** Tao Li, Jing Chen

**Affiliations:** 1 Department of Physical Medicine and Rehabilitation, Peking University People’s Hospital, Beijing, CHN; 2 Department of Rehabilitation Medicine, Singapore General Hospital, Singapore, SGP

**Keywords:** clinical applications, neural plasticity, neuromodulation, spinal cord injuries, spinal cord stimulation

## Abstract

Spinal cord injury (SCI) represents a significant medical challenge, leading to profound and often debilitating neurological deficits that adversely affect motor, sensory, and autonomic functions. Traditional rehabilitation strategies, while essential in the management of SCI, often exhibit limited efficacy in restoring lost functions, leaving many individuals with permanent disabilities. In this context, spinal cord stimulation (SCS) emerges as a novel and promising therapeutic approach with the potential to enhance neurological recovery by promoting neural plasticity and activating residual neural pathways. This narrative review provides a comprehensive examination of SCS, elucidating its underlying mechanisms of action, technological advancements, clinical applications, and associated outcomes in patients with SCI. Both invasive (epidural) and noninvasive (transcutaneous) SCS are discussed, emphasizing their therapeutic potentials with current established evidence. This narrative review integrates findings from preclinical and clinical studies, highlighting the role of SCS in facilitating functional recovery. Furthermore, this review highlights the challenges faced in the field, including variability in patient responses, lack of standardized stimulation protocols, and the need for further research to substantiate long-term outcomes. We conclude by discussing future directions for SCS research, including the development of closed-loop systems and innovative brain-spine interfaces, which may optimize treatment delivery and enhance functional recovery in individuals with SCI.

## Introduction and background

Spinal cord injury (SCI) is a complex condition that arises from either traumatic events, such as motor vehicle accidents or falls, or nontraumatic causes, such as vascular accidents, infections, tumors, or degenerative diseases. SCI affects individuals across all age groups but has a disproportionately high impact on younger populations, leading to significant socioeconomic and psychological consequences [1]. SCI frequently results in permanent loss of sensorimotor and autonomic function, significantly impacting functional independence, quality of life, and physical and psychological health [2]. SCI often results in the irreversible loss of motor, sensory, and autonomic functions, posing substantial challenges for affected individuals, their families, and the healthcare systems. This contributes to significant socioeconomic challenges, as individuals face reduced productivity, increased healthcare costs, and long-term dependency on caregivers. Additionally, SCI is associated with substantial psychological burdens, including depression and anxiety, due to the sudden and dramatic change in lifestyle and independence.

Traditional rehabilitation management of SCI has primarily focused on minimizing secondary complications such as pressure injuries, urinary tract infections, bowel dysfunction, and spasticity. Additionally, it aims to optimize residual function through therapies designed to enhance mobility, strength, sensation, and coordination, while also incorporating compensatory strategies to address functional loss. Although these approaches provide important benefits, they are often insufficient to restore or compensate for substantial functional impairments [3]. In recent years, advances in neuromodulation have opened up new possibilities for addressing the challenges of SCI. Spinal cord stimulation (SCS), in particular, has emerged as a promising approach to promote neurological recovery. By promoting neuroplasticity and reactivating residual neural pathways, SCS has demonstrated the potential to enable functional improvements that were once thought unattainable. Increasing evidence suggests that patients with chronic SCI can experience meaningful neurological and functional gains with SCS, offering renewed hope for functional restoration and improved quality of life. 

This review explores the application of SCS for neurological recovery in SCI, with a focus on understanding its mechanisms, evaluating clinical evidence, and identifying future directions for research and practice. Both invasive techniques, such as epidural spinal cord stimulation (eSCS), and noninvasive methods, such as transcutaneous spinal cord stimulation (tSCS), are discussed. The therapeutic potential of SCS is critically examined, along with the challenges and opportunities involved in integrating these advanced interventions into routine clinical practice.

## Review

Pathophysiology after traumatic SCI

The pathophysiological progression of traumatic SCI involves a complex cascade of events that can be broadly divided into primary and secondary phases [4,5]. These processes contribute to the extensive and often irreversible damage in SCI, ultimately impairing functional recovery. 

The primary phase starts immediately following the injury and is the direct result of mechanical forces such as compression, contusion, or laceration. These forces lead to involve mechanical damage to the spinal tissues, blood vessels, and the blood-spinal cord barrier (BSCB) [6]. The breakdown of the BSCB results in increased vascular permeability, allowing harmful substances to infiltrate the cord. This phase is characterized by hemorrhage, ischemia, and direct neuronal death, which collectively initiate a cascade of biochemical and cellular responses [7,8]. 

The secondary phase begins minutes to hours after the initial injury and continues for days, weeks, or even months. It is marked by a series of deleterious processes that extend the damage, including inflammation, oxidative stress, and excitotoxicity, which exacerbate the initial damage. The inflammatory response causes tissue swelling, leading to compression of nearby structures and further ischemia. Elevated intrathecal pressure can impair the cerebrospinal fluid (CSF) flow, compounding the damage. Activated astrocytes and microglia proliferate around the lesion site, forming a glial scar. While initially protective, this scar ultimately inhibits axonal regeneration, creating physical and biochemical barriers to neural repair [5,8,9]. Over time, necrotic tissue at the injury site is replaced by fluid-filled cystic cavities, creating physical gaps that block neural reconnection. These cavities represent a major challenge for therapeutic interventions aimed at restoring connectivity. The forming of syringomyelia and progressive neurodegeneration, further impede functional recovery [10,11]. 

Spinal cord stimulation (SCS) in SCI

SCS represents a promising therapeutic approach that provides electric current to stimulate the spinal cord either epidurally or transcutaneously. Following early reports of volitional movement recovery in patients with motor-complete SCI [12], SCS has gained significant attention for its potential in facilitating neurological recovery, and numerous studies examining both epidural and tSCS have demonstrated its effectiveness in neurological recovery following SCI [13-17]. 

SCS can be broadly categorized into closed-loop and open-loop stimulation [18]. Open-loop systems deliver constant or cyclic stimulation irrespective of real-time sensory or motor feedback. In contrast, closed-loop systems dynamically adjust stimulation parameters based on real-time data, such as gait cycles or brain activity, to achieve more targeted and efficient neuromodulation.

Different SCS studies employ various technological strategies, including different sites of electrical stimulation, electrode designs, stimulation parameters, and temporal patterns [16,19]. Despite its crucial role, the actual mechanisms of SCS and the association between technological variables and physiological mechanisms are not yet fully understood [2]. 

Mechanisms of action in SCS

Enhancement of Monosynaptic Reflex Activation

The predominant theory suggests that SCS activates large-to-medium afferent fibers, thereby enhancing the interneural connections between sensory afferents and motoneurons [18,20-22]. Ia fibers transmit information about muscle length, velocity, and force development, which are essential for estimating limb position and other movement dynamics. According to the classical cable theory, large-diameter myelinated axons are particularly excitable in response to electrical currents [23]. SCS strengthens monosynaptic reflex activation onto motoneurons that control agonist muscles and facilitates interneuronal circuits within motor pools, both vital for locomotor recovery following SCI [24,25].

Numerous studies support this proposed mechanism [24,26]. For example, animal models experiencing loss of proprioceptive afferents fail to regain control of hindlimbs and show maladaptive reorganization of descending circuitry [24,26]. Proprioceptive ablation results in a regression of sensorimotor improvements in SCI animal models [26]. 

Reorganization of Spinal Networks

Research indicates that spinal cord circuits, even when disconnected from the brain after complete spinal transection, can independently process somatosensory information and adapt motor outputs [24]. Complex intraneuronal networks connect different neuron clusters, spinal segments, and hemicords, facilitating effective signal integration and motor coordination [2,27]. 

Spinal neural networks can generate rhythmic movements without direct input from the brain or sensory feedback [28,29]. SCS, when paired with physical training, is thought to reorganize propriospinal circuitry around the lesion site and engage central pattern generators (CPGs), which are neural circuits responsible for rhythmic outputs [12]. Candidate CPG neurons have been localized near the central canal and within the medial intermediate zone of the lumbar spinal segments, highlighting their link to locomotor rhythms [30]. 

Evidence suggests that SCS can indirectly recruit and modulate these circuits, reorganizing propriospinal pathways to enhance rhythmic activity and hindlimb coordination [25,31]. The lumbar spinal cord’s CPGs enable the rhythmic activation of flexor and extensor muscles, crucial for walking recovery with SCS [32]. 

Strengthening Response to Supraspinal Inputs

SCS also enhances motoneuron excitability to descending supraspinal inputs, including corticospinal, reticulospinal, and rubrospinal tracts. These pathways are critical for restoring voluntary movement control [18], as they contribute to sensory prediction, real-time adjustments, and motor memory [25]. Despite severe SCI, some anatomical continuity within the spinal cord often remains intact, even in clinically complete injuries [18,33]. Continuous SCS may increase motoneurons' susceptibility to depolarization from remaining supraspinal inputs, mediating corticospinal plasticity that augments muscle coordination and movement error correction [34].

**Figure 1:**
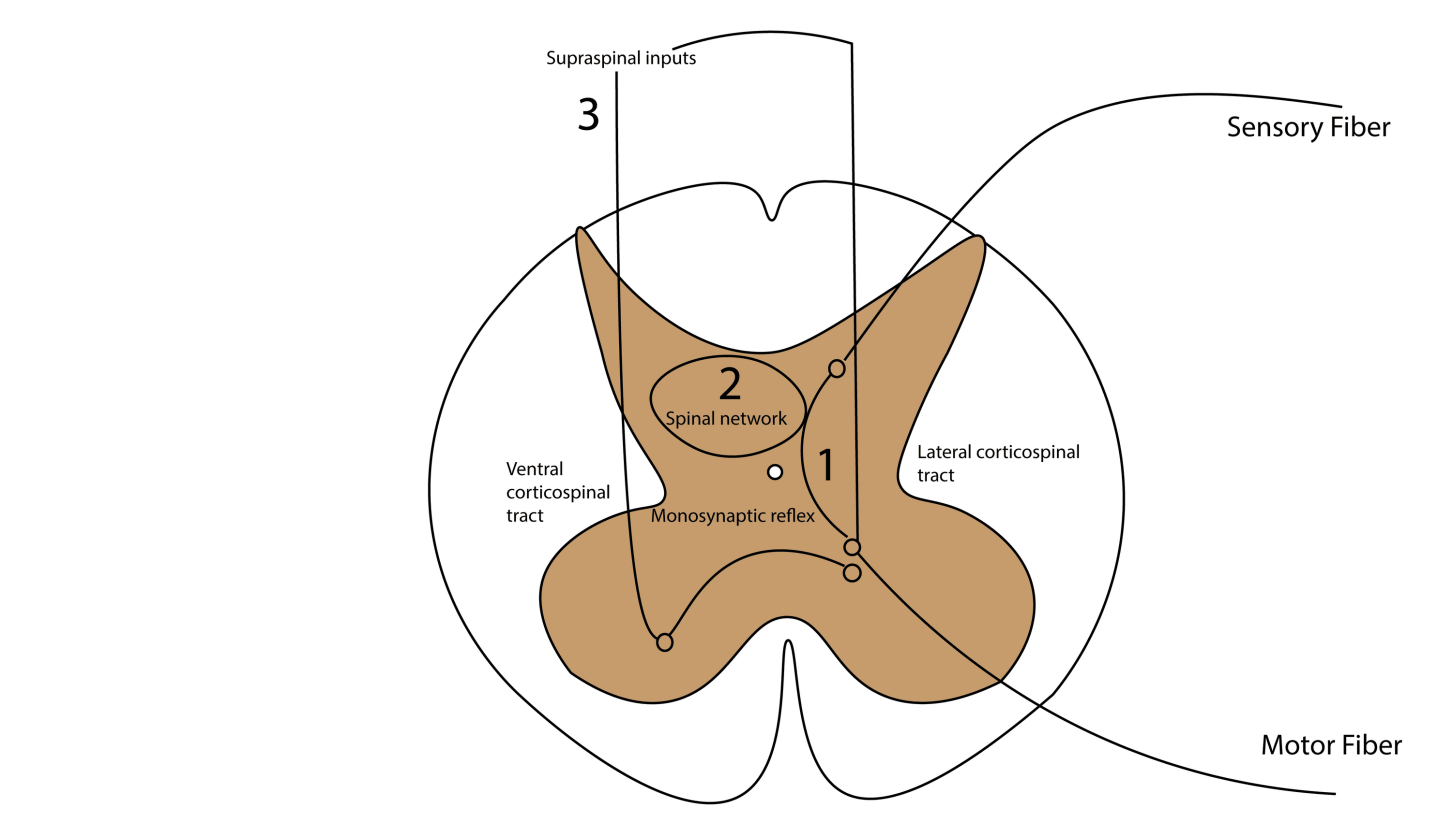
Mechanisms of action in SCS SCS: spinal cord stimulation; SCI: spinal cord injury The proposed mechanisms of SCS in SCI include (1) enhancement of monosynaptic reflex activation, (2) reorganization of spinal networks, and (3) strengthening response to supraspinal inputs (this figure was produced by the authors)

Several studies validate this hypothesis. For instance, Guiho et al. found that tSCS facilitated supraspinal-evoked responses in monkeys [35]. Additionally, Parhizi et al. reported that simultaneous cervical and lumbar tSCS increased activation of corticospinal pathways in humans [36]. The proposed mechanisms are summarized in Figure 1.

Epidural spinal cord stimulation (eSCS)

eSCS Settings and Parameters

eSCS was originally developed primarily for the management of pain. Over time, this technique has gained increasing attention for its potential to facilitate neurological recovery in individuals with SCI [14]. By delivering electrical stimulation through implanted electrodes, eSCS can activate spinal circuits and facilitate recovery in motor, sensory, and autonomic domains. Typically, multielectrode arrays are placed between the L1 and S1 spinal cord segments [37]. To enhance its effectiveness, paddle leads are positioned over the dorsal root entry zones within the lumbar spinal cord [16]. Accurate electrode placement plays a fundamental role in the effectiveness of eSCS, as distinct spinal cord regions are responsible for different body functions. As such, careful preoperative assessments are essential for optimizing treatment outcomes. 

Candidates for eSCS often undergo comprehensive preimplantation evaluations, such as magnetic resonance imaging (MRI) of the spine. MRI is pivotal in creating a neuroanatomical 3D reconstruction of the spinal cord to increase the precision of electrode placement [38]. Moreover, MRI measurements of spared spinal cord tissue have been found to be significantly correlated with standing outcomes with eSCS [39]. Recent studies by Lorach et al. emphasized the importance of utilizing personalized models of the spine elaborated from high-resolution structural imaging [40]. Notably, the study demonstrates that combining eSCS with intense neurorehabilitation can improve multiorgan function in patients with acute and chronic, motor-complete paralysis (American Spinal Injury Association Impairment Scale (AIS) A) in both clinical and home settings. Within eight weeks, eight patients' AIS scores improved from A to C, and two patients' AIS scores changed from A to D. Six patients experienced a ≥1-segment improvement in injury functional levels. All patients showed significant gains in lower limb muscle strength and greater walking independence [41].

Once the electrodes are implanted, the stimulation parameters are adjusted and tailored based on the patient’s response and the specific rehabilitation goals of the patient. Typically, higher segments target volitional control and cardiovascular functions, and lower segments focus on urogenital function [14]. Minassian et al. have demonstrated that different frequencies of stimulation can influence muscle activation patterns in the lower limbs, with 5-15 Hz stimulation initiating lower limb extension and 25-50 Hz eliciting alternating lower limb flexion/extension in supine individuals [42]. Stimulation parameters vary significantly across studies [14], with frequencies ranging from 0.2 to 400 Hz, pulse widths between 150 and 1000 microseconds (µs), and amplitudes spanning 0.1 to 40 V or 0.1 to 15 mA [14].

Clinical Evidence of eSCS

Numerous studies combining eSCS with activity-based therapy have demonstrated significant improvements [12,16,19,39-41,43-45]. The key eSCS studies are summarized in Table 1. 

**Table 1:** Summary of key eSCS studies in SCI SCS: spinal cord stimulation; eSCS: epidural spinal cord stimulation; SCI: spinal cord injury; SBP: systolic blood pressure;

Characteristics of eSCS for motor control in upper limbs											
Author (year) [citation]	No. of participants	AIS classification	Injury duration (yr)	No. of channels	Stimulation parameters					Lead placement	Main findings
					Frequency (Hz)		Pulse width (us)		Amplitude		
Lu et al. (2016) [43]	2	B	2-2.5	16	2-40 Hz	210		0.1-10.0 mA		C4/C5-T1	Cervical cord neuromodulation improves volitional hand motor function (grip and control) in individuals with chronic tetraplegia
Characteristics of SCS for motor control in lower limbs											
Author (year) [citation]	No. of participants	AIS classification	Injury duration (yr)	No. of channels	Stimulation parameters					Lead placement	Main findings
					Frequency (Hz)		Pulse width (us)		Amplitude		
Minassian et al. (2007) [42]	15	A	Not reported	4	2.2-50 Hz	210		1-10 V		T10-L1	5-15 Hz stimulation initiates lower limb extension; 25-50 Hz elicits alternating lower limb flexion/extension in supine individuals
Harkema et al. (2011) [12]	1	B	3.4	16	5-40 Hz	210 or 450		0.5-10 V		Paddle (L1-S1)	15 Hz stimulation was optimized for standing, and 30-40 Hz for stepping. Recovery of supraspinal control of some leg movements occurred after 7 months, but only during eSCS
Angeli et al. (2014) [19]	4	A-B	2.2-4.2	16	25-30 Hz	Not reported		Not reported		Paddle (L1-S1)	eSCS enables patients with complete paralysis to process conceptual, auditory, and visual input to regain relatively fine voluntary motor control of paralyzed muscles
Danner et al. (2015) [46]	10	A-B	2-8	4	2-130 Hz	210		0-10.5 V		T11-L1	Rhythmic activity was generated in subjects after stimulation
Hofstoetter et al. (2015) [47]	8	A-B	1-13	4	2-130 Hz	210		0-10.5 V		T11-L1	Repeated epidural stimulation of the lumbosacral spinal cord can generate rhythmic burst-like activity at 20-60 Hz
Wagner et al. (2018) [17]	3	C-D	4-6	16	20-129 Hz	Not reported		0.6-8 mA		Paddle (T11-L1)	eSCS re-established adaptive control of paralyzed muscles during overground walking within one week; spatiotemporal stimulation led to volitional control over walking and cycling
Rowald et al. (2022) [16]	3	A-B	1.3-8.9	16	20 or 100 Hz	500		0.5 V		Paddle (T12-S2)	Activity-specific eSCS enabled standing, swimming, cycling, walking, and control of trunk movements within 1 day; gait improvement and volitional motor control also occurred after 1 week post-eSCS. Neurorehabilitation mediated the restoration of these locomotor activities in community settings
Kandhari et al. (2022) [41]	10	A	0.3-2	16	15-60 Hz	210-400		1-6 V		Paddle (T11-L1)	AIS scores changed from A-C for 8 patients and A-D for 2 patients after 8 weeks, with 6 patients improving their functional level of injury by ≥1 segment. Significant improvements in lower extremity muscles were seen in all patients. Independence and comfort were seen during walking post–therapy
Smith et al. (2022) [39]	11	A-B	2-9	16	Not reported	Not reported		Not reported		Paddle (lumbosacral)	Measures of spared spinal cord tissue significantly relate to standing outcomes with eSCS. 7/11 subjects with spared spinal cord tissue achieved some knee independence
Lorach et al. (2023) [40]	1	Not reported	10	16	40 Hz	300		14-16 mA		Paddle (T11-L1)	The author emphasized the importance of utilizing a personalized model of the spine elaborated from high-resolution structural imaging
Characteristics of SCS for autonomic controls											
Author (year)[citation]	No. of participants	AIS classification	Injury duration (yr)	No. of channels	Stimulation parameters					Lead placement	Main findings
					Frequency (Hz)		Pulse width (us)		Amplitude		
Singh Sahni et al. (1991) [48]	33	A-D	0.58-31.5	4	25-130	180-450		0.25-10.5 V		Paddle	Postoperative changes in the lower urinary tract function were noted in 6 patients. Urodynamic parameters did not change significantly following implantation in the remaining 17 patients
DiMarco et al. (2009) [49]	9	Not reported	1-34	1	30-40 Hz	150-200		30-40 V		T9, T11, L1	Supramaximal SCS led to increases in both mean maximum peak airflow rates and airway pressure
Hofstoetter et al. (2015) [47]	8	A-B	1-13	4	2-130 Hz	210		0-10.5 V		T11-L1	Repeated epidural stimulation of the lumbosacral spinal cord can generate rhythmic burst-like activity at 20-60 Hz
Harkema et al. (2018) [50]	4	Not reported	Chronic	1	Not reported	Not reported		Not reported		Paddle	eSCS provides sustained improvement in orthostatic hypotension in cervical chronic SCI
Beck et al. (2021) [51]	2	A	>3	16	Not reported	Not reported		Not reported		Paddle (T12-L1)	eSCS optimized for locomotion negatively impacted neurogenic bladder functionality, leading to an increase in episodes of urinary incontinence with worsening bladder compliance and pressures. One participant showed an increase in lean body mass
Boakye et al. (2023) [52]	25	A-B	2-17	16	2 Hz	450		Increased from 0.1 to 0.5 V with 0.1 V		Paddle (T12-L1)	All participants achieved SBP regulation within 110-120 mmHg and were able to integrate the eSCS into their daily lives

Most of the studies are done in motor-complete SCI to improve motor function in the lower limbs [12,16,19,42,45-47]. For instance, participants with motor-complete paraplegia who underwent 80 sessions of activity-based training, each lasting 60 minutes, coupled with eSCS, demonstrated the ability to stand with minimal assistance for up to five minutes [12]. Beyond these immediate improvements, Luz et al. reported that high-intensity task-specific training, consisting of four to five two-hour sessions per week, facilitated substantial neurological gains over five months of weight-supported overground training. This led to improvements in motor scores for all participants, with some achieving voluntary control of previously paralyzed muscles even in the absence of stimulation. Notably, motor scores for all participants improved, and some achieved voluntary control of paralyzed muscles even without stimulation [45]. 

While robust evidence supports lower limb recovery, the efficacy of eSCS in improving upper limb function remains limited. Preclinical studies in cervical SCI models have shown positive effects, but clinical evidence remains confined to case reports [43]. For example, two patients with a motor-complete SCI (AIS B) underwent implantation of 16 electrode arrays at the C5 to T1 level. Stimulation parameters include frequencies of 2 to 40 Hz, an amplitude of 0.1 to 10 mA, and a pulse width of 210 µs. Both participants showed significant functional gains, with upper limb motor scores improving by 16 to 23 points. Functional assessments using the Spinal Cord Independence Measure (SCIM) corroborated these improvements, indicating enhanced independence in daily activities [43]. 

The therapeutic benefits of eSCS extend beyond motor recovery. Studies have demonstrated its positive impact on autonomic functions, including bowel and bladder regulation, spasticity reduction, respiratory function, and cardiovascular stability [48,49,51,52]. Boakye et al. reported that eSCS can be a promising option for improving quality of life [52]. However, optimizing stimulation for motor control may negatively impact bladder function. For instance, Beck et al. demonstrated that motor-focused stimulation parameters could compromise bladder outcomes, indicating the need for tailored protocols [51]. 

Transcutaneous spinal cord stimulation (tSCS)

tSCS offers a noninvasive alternative by delivering electrical currents through surface electrodes aligned with the vertebral columns. The stimulation targets dorsal roots and interneurons to lower the activation threshold of motor neurons. By activating these regions, tSCS modulates spinal reflexes, enhances motor neuron excitability, and promotes communication between intact spinal networks below the injury site. 

tSCS Parameters

The efficacy of tSCS depends on various parameters, including electrode placement, electrode size, waveform, stimulus intensity, pulse width, and frequency.

Two types of electrodes are utilized in tSCS-negative electrode (cathode) and positive electrode (anode). Cathode placement is determined by the motor control region. This strategy is built on the premise that targeting the cervical and lumbar enlargements allows the stimulation of a large number of neural circuits responsible for muscle activation in those particular regions [53]. Typically, for upper limb stimulation, cathodes are placed between the C3/C4 and C6/C7 spinous processes [54]. For the lower limbs, the cathodes are typically placed between the T10 and L2 spinous processes [53]. Anodes are usually positioned bilaterally over the bilateral iliac crests but may also be placed on the lower abdomen, or clavicles [53,54]. Skiadopoulos et al. investigated the physiological differences of the cathodal electrode arranged in different settings and concluded that a rectangular cathode electrode placed at the midline requires less current to produce trans-spinal evoked potentials and maximizes spinal inhibition [55]. However, there remains no consensus on the optimal electrode sizes or configurations, nor on whether single-site or multi-site stimulation is more effective [53]. 

tSCS can utilize both biphasic and monophasic waveforms [53], although limited comparative studies exist. One study indicated that the average stimulation intensity was higher in the biphasic group [54], possibly due to its absence of an electrochemical polarization effect, which might allow higher stimulation levels while reducing the risk of tissue damage [56]. Stimulation amplitudes vary widely, from submotor to supramotor thresholds [57-59]. For the upper limbs, the motor threshold is approximately 90-100 mA, while lower limbs require higher thresholds, ranging from 100 to 180 mA. Adjusting amplitudes incrementally based on participant tolerance or observable motor improvements, as demonstrated by Gelenitis et al., has proven effective in achieving positive results [54]. 

Pulse widths ranging from 0.5 ms to 2 ms are used in tSCS, with 1 ms being the most commonly used and favored [53,54]. Stimulation frequencies to elicit motor responses generally range from 30 to 50 Hz [53], with higher frequencies noted for their role in reducing spasticity [59]. An emerging trend involves using a carrier frequency alongside with burst frequency, as this approach has been reported to improve muscle strength while minimizing discomfort [57,60]. 

Despite ongoing research, standardized tSCS parameters have yet to be established, necessitating further investigation to optimize therapeutic outcomes and ensure consistency across clinical applications. 

Clinical Evidence of tSCS for Functional Recovery

tSCS has shown significant potential in improving motor and autonomic functions in individuals with SCI. Its applications span from enhancing voluntary movement to addressing disruptions in autonomic regulation, with growing evidence supporting its efficacy in both. The key tSCS studies are summarized in Table 2.

**Table 2:** Summary of key tSCS studies in SCI tSCS: transcutaneous spinal cord stimulation; SCI: spinal cord injury; AIS: American Spinal Injury Association Impairment Scale

Characteristics of tSCS for motor control in the upper limbs										
Author (year) [citation]	No. of participants	AIS classification	Injury duration (yr)	No. of channels	Stimulation parameters			Cathode locations	Anode locations	Main findings
					Frequency (Hz)	Pulse width (us)	Amplitude			
Gad et al. (2018) [61]	6	B-C	1-21	2	30 Hz with a carrier frequency of 10 kHz	1000	10-250 mA	C3/4, C6/7	Bilateral iliac crests	Improvement in voluntary hand function
Sayenko et al. (2019) [62]	15	A-C	2-13	2	30 Hz with a carrier frequency of 10 kHz	1000	Up to 200 mA	T11/T12, L1/L2	Bilateral iliac crests	With stimulation, all participants could maintain upright standing
Wu et al.(2020) [56]	13	B-D	1-20	1	0.2 Hz	2000	102 mA	C4/5 anterior, T2-4 posterior	Distal clavicles	Cervical tSCS easily activates upper limb muscles
Benavides et al. (2020) [63]	17	A-D	4-19	1	30 Hz with a carrier frequency of 5 kHz	200	55 -95 mA	C5/6	Bilateral iliac crests	Hand and arm function improved largely with tSCS
Inanici et al. (2021) [64]	6	B-D	1.5-12	2	30 Hz with a 10 kHz carrier frequency	1000	40-90 mA	Above and below the injury	Bilateral anterior iliac crests	tSCS restores movement and function in the upper limbs
Moritz et al. (2024) [13]	60	B-D	>1	2	30 Hz with a carrier frequency of 10 kHz	1000	Not fixed, based on motor performance	C3/4, C6/7	Bilateral anterior superior iliac crests	The study demonstrates the safety and efficacy of tSCS in improving hand and arm functions in cervical SCI
Characteristics of tSCS for motor control in the lower limbs										
Author (year) [citation]	No. of participants	AIS classification	Time since injury (years)	No. of stimulation sites	Stimulation parameters			Cathode locations	Anode locations	Main findings
					Frequency	Pulse width (us)	Amplitude			
Hofstoetter et al. (2015) [65]	3	D	9-12	1	30 Hz	Not specified	27 V	T11/T12 paraspinally	Paraumbilically	tSCS can be used as an electrical neuroprosthesis augmenting remaining motor control
Gerasimenko et al. (2015) [66]	5	A-B	2-6	1	30 Hz or 5 Hz with a carrier frequency of 10 kHz	Not specified	Submotor	T11 or coccyx 1	Iliac crest	tSCS facilitates voluntary movement
Minassian et al. (2016) [44]	4	A	1.7-4.8	1	30 Hz	Not specified	100-170 mA	T11 and T12	abdomen	Adding tSCS increased muscle activity
Sayenko et al. (2019) [62]	15	A-C	2-13	2	15-30 Hz with a carrier frequency of 10 kHz	1000	up to 150 mA	T11, L1	Bilateral iliac crests	Self-assisted standing improved
Shapkova et al. (2020) [59]	19	A-C	1.5-11	1	Not specified	500	unspecified	T12	Central abdomen	tSCS may facilitate training and walking in the exoskeleton
McHugh et al. (2020) [67]	10	C-D	2-57	1	50 Hz	1000	20-80	T11/12	Lower abdomen	tSCS is feasible and useful as an adjunct to walking-based therapy.
Al’joboori et al. (2020) [58]	9	A-D	1-9	1	30 Hz	1000	110mA	T10/11	T12/L1	tSCS improved the lower limbs' motor control
Sutor et al. (2022) [68]	8	A-C	2-21	2	30 Hz	1000	Not specified	L1-S2	Bilateral iliac crest	Preliminary positive data on tSCS with exoskeleton-assisted walking
Samejima et al. (2022) [57]	2	D	3.5-4.5	4	30 Hz with a carrier frequency of 10 kHz	1000	5-40 mA at C3/4, C6/7, 35-75 mA at T11, L1	C3/4, C6/7, T11, L1	Bilateral iliac crests	tSCS improved walking distance
Characteristics of tSCS for autonomic controls										
Author (year) [citation]	No. of participants	AIS classification	Injury duration	No. of leads	Stimulation parameters			Cathode locations	Anode locations	Main findings
					Frequency	Pulse width (us)	Amplitude			
Phillips et al. (2018) [69]	5	A-B	>3	1	30 Hz	1000	10-70 mA	T7/8	Bilateral iliac crest	Orthostatic hypotension improved
Sachdeva et al. (2021) [70]	1	A-B	3	1	30 Hz	2000	20-30 mA	T7/8	Bilateral iliac crest	Autonomic dysreflexia reduced
Solinsky et al. (2023) [71]	4	A	Chronic	1	30-120 Hz, with a carrier frequency of 5 kHz	Not specified	80% of the motor threshold	T10/11	Bilateral iliac crests	tSCS generates sympathetic activation and may lower the threshold for autonomic dysreflexia
Gad et al. (2018) [72]	7	A-C	1-23	1	0.5 Hz and 30 Hz	Not specified	10-200	T11/12, L1/2	Bilateral iliac crests	tSCS improved bladder function
Kreydin et al. (2020) [73]	5	A-C	1.5-11	1	30 Hz with a carrier frequency of 10 kHz	1000	Submotor	T11-L1	Bilateral iliac crests	tSCS improved symptoms of overactive bladder
Samejima et al. (2022) [57]	2	D	3.5-4.5	4	30 Hz with a carrier frequency of 10 kHz	1000	5-40 mA at C3/4, C6/7, 35-75 mA at T11, L1	C3/4, C6/7, T11, L1	Bilateral iliac crests	tSCS improved bowel and bladder function

tSCS has demonstrated remarkable benefits in individuals with incomplete SCI. A systematic review by Megía García et al. encompassing 13 studies with 55 participants highlighted significant improvements in motor responses, voluntary movements, muscle strength, and overall functionality [74]. When combined with activity-based rehabilitation, tSCS has been shown to improve voluntary motor control, standing, and walking capabilities [57,59,67,68]. 

In individuals with tetraplegia, tSCS targeting the cervical spine has shown promise in improving upper limb motor control [13,24,56,59,62-64]. Enhanced grip strength, better arm movement, and improved fine motor skills have been reported when tSCS is administered to the cervical spine [13]. These outcomes are particularly significant for individuals with tetraplegia, where even small improvements in upper limb function can greatly enhance independence and quality of life. 

Beyond motor enhancements, tSCS has demonstrated efficacy in addressing autonomic functions associated with SCI. These include bladder and bowel control and blood pressure regulation (Table 2). Several case series reported that tSCS improved overactive bladder symptoms as well as urodynamic findings [72,73]. Samejima et al. reported tSCS improved both bladder and bowel function [57]. Additionally, tSCS has been associated with improved blood pressure stability in individuals with orthostatic hypotension post-SCI, though it remains unclear whether this effect reflects restored autonomic regulation or a compensatory pathological reflex [75]. While these findings are encouraging, variability in study design and outcomes highlights the need for higher-quality research. 

Contraindications, adverse effects, and safety in SCS

eSCS involves surgical implantation of a spinal cord stimulator epidurally. Due to its invasive nature, in most studies, eSCS is generally introduced in cases of motor-complete SCI at least one-year post-injury, when spontaneous recovery through conventional measures is deemed unlikely [12,16,17,40]. The risks associated with eSCS are well-documented in treating patients with chronic pain [76]. These include the risks related to surgical procedures, such as infection, hematoma, CSF leak, and nerve injury, as well as device-related complications, including electrode migration, malfunction of electrode wires, premature battery depletion, epidural electrode encapsulation, skin erosion, and pain [77]. In patients with SCI, the safety profile of eSCS is similar to eSCS for chronic pain treatment. The most frequently reported adverse effects are infection, followed by pain, unusual sensation, hardware failure, lead migration, pain, and the need for surgical implant revision [78]. 

In contrast, tSCS is a noninvasive alternative that broadens the scope of applications. It is most commonly employed in patients with incomplete SCI across a range of severities, from AIS B to AIS D [13,53,54]. According to a systematic review by Megía García et al. [74], tSCS is generally well-tolerated, with cutaneous irritation from repeated stimulation being the primary adverse effect reported. Manufacturer-reported contraindications for tSCS include the presence of swollen, infected, inflamed, or damaged skin at the electrode application sites, the presence of cancerous lesions near the treatment area, and patients with active implanted medical device or wearable defibrillators. Relative contraindications include severe and uncontrolled autonomic dysreflexia, unstable medical conditions that interfere with tSCS, and pregnancy [54]. Adverse events associated with tSCS are rare and include skin breakdown, unpleasant sensations, increased spasticity, intervention intolerance, and allergic reactions to skin adhesives [78]. 

Challenges and future directions

While the evidence supporting SCS as a tool for neural restoration in SCI is promising, several challenges remain. One of the most significant challenges in the use of SCS is the variability in patient response. Not all individuals with SCI derive equal benefit from SCS, and the factors underlying this variability are not fully understood. Differences in injury severity, chronicity, the specific level and location of the injury, and patient-specific anatomical and physiological characteristics likely contribute to these disparities. For instance, individuals with incomplete injuries or preserved neural pathways may respond more favorably compared to those with complete injuries. Identifying predictors of responsiveness through advanced imaging, electrophysiological studies, and biomarker analysis could guide patient selection and help tailor interventions to individual needs. 

Another major limitation is the lack of standardized protocols for the application of SCS in SCI rehabilitation. Current studies use diverse stimulation parameters, including varying frequencies, intensities, electrode placements, and session durations. This lack of consistency complicates the interpretation of study results and hinders the ability to establish best practices for clinical use. Developing evidence-based, standardized protocols is crucial for optimizing outcomes. Moreover, it will be important to assess how different parameters influence specific goals, such as motor recovery, autonomic regulation, or pain reduction, to refine individualized treatment strategies.

While many studies have reported short-term improvements in motor and autonomic functions, the sustainability of these gains remains uncertain. The potential for lasting benefits after discontinuing SCS is critical for its translation into meaningful clinical interventions. Understanding the mechanisms underlying both the immediate- and long-term effects of stimulation is a research priority. Additionally, combining SCS with adjunct therapies, such as activity-based rehabilitation, pharmacological agents, or regenerative approaches like stem cell therapy, may help consolidate and sustain functional improvements.

Most of the existing evidence on SCS comes from small-scale, exploratory studies. While these have provided valuable insights, their limited sample sizes and methodological heterogeneity reduce the generalizability of findings to the broader SCI population. Larger, multicenter randomized controlled trials (RCTs) are essential to establish the efficacy, safety, and cost-effectiveness of SCS. Furthermore, long-term follow-up studies will be needed to assess the durability of functional gains and the potential impact on quality of life and independence.

Future directions include the development of closed-loop SCS systems, which trigger or modulate SCS in response to movement information. These systems could be paired with artificial intelligence algorithms to develop more precise and individualized stimulation programs. Incorporating brain-spine interfaces to rebuild the connection between the brain and the spinal cord could provide more precise stimulation and improve clinical outcomes [40]. SCS is an area with significant potential and is poised to significantly impact the field of SCI rehabilitation. 

## Conclusions

SCS has emerged as a groundbreaking therapeutic modality for promoting neurological recovery in SCI. By targeting residual neural pathways and enhancing neuroplasticity, SCS offers renewed hope for individuals who face the devastating consequences of SCI. Both invasive approaches and noninvasive alternatives of SCS have demonstrated significant potential in facilitating motor recovery, improving autonomic functions, and enhancing quality of life. Despite the promising advancements, challenges remain in translating SCS into routine clinical practice. Variability in patient response, lack of standardized stimulation protocols, and questions surrounding the sustainability of functional gains highlight the need for further research. Additionally, small-scale exploratory studies have been invaluable for understanding the potential of SCS, but larger RCTs are essential to establish its efficacy, safety, and generalizability across diverse patient populations.

SCS represents a significant paradigm shift in SCI rehabilitation, offering the potential to restore sensorimotor and autonomic functions previously deemed unattainable. While the road to widespread clinical adoption is fraught with challenges, ongoing research and technological advancements are likely to unlock the full potential of SCS. As the field continues to evolve, SCS stands poised to transform the lives of individuals with SCI, providing them with renewed hope for independence, functionality, and an improved quality of life.
